# [Corrigendum] Resveratrol upregulates SOCS1 production by lipopolysaccharide-stimulated RAW264.7 macrophages by inhibiting miR-155

**DOI:** 10.3892/ijmm.2024.5386

**Published:** 2024-06-03

**Authors:** Chunfang Ma, Yin Wang, Aijuan Shen, Wanru Cai

Int J Mol Med 39: 231-237, 2017; DOI: 10.3892/ijmm.2016.2802

Following the publication of the above article, an interested reader drew to the authors' attention that the gel slice shown for the p38MAPK bands in Fig. 2C on p. 234 was strikingly similar to the β-actin bands shown in Fig. 3B on p. 235, albeit their orientations appeared to have been altered horizontally through 180°. The authors consulted their original data, and were able to determine that the duplication of these figure parts had inadvertently arisen during the process of compiling Fig. 2. The revised version of Fig. 2, featuring the correct p38MAPK data in Fig. 2C, is shown on the next page. The authors confirm that the error associated with this figure did not have any significant impact on either the results or the conclusions reported in this study, and are grateful to the Editor of *International Journal of Molecular Medicine* for allowing them the opportunity to publish this Corrigendum. Furthermore, they apologize to the readership of the Journal for any inconvenience caused.

## Figures and Tables

**Figure 2:**
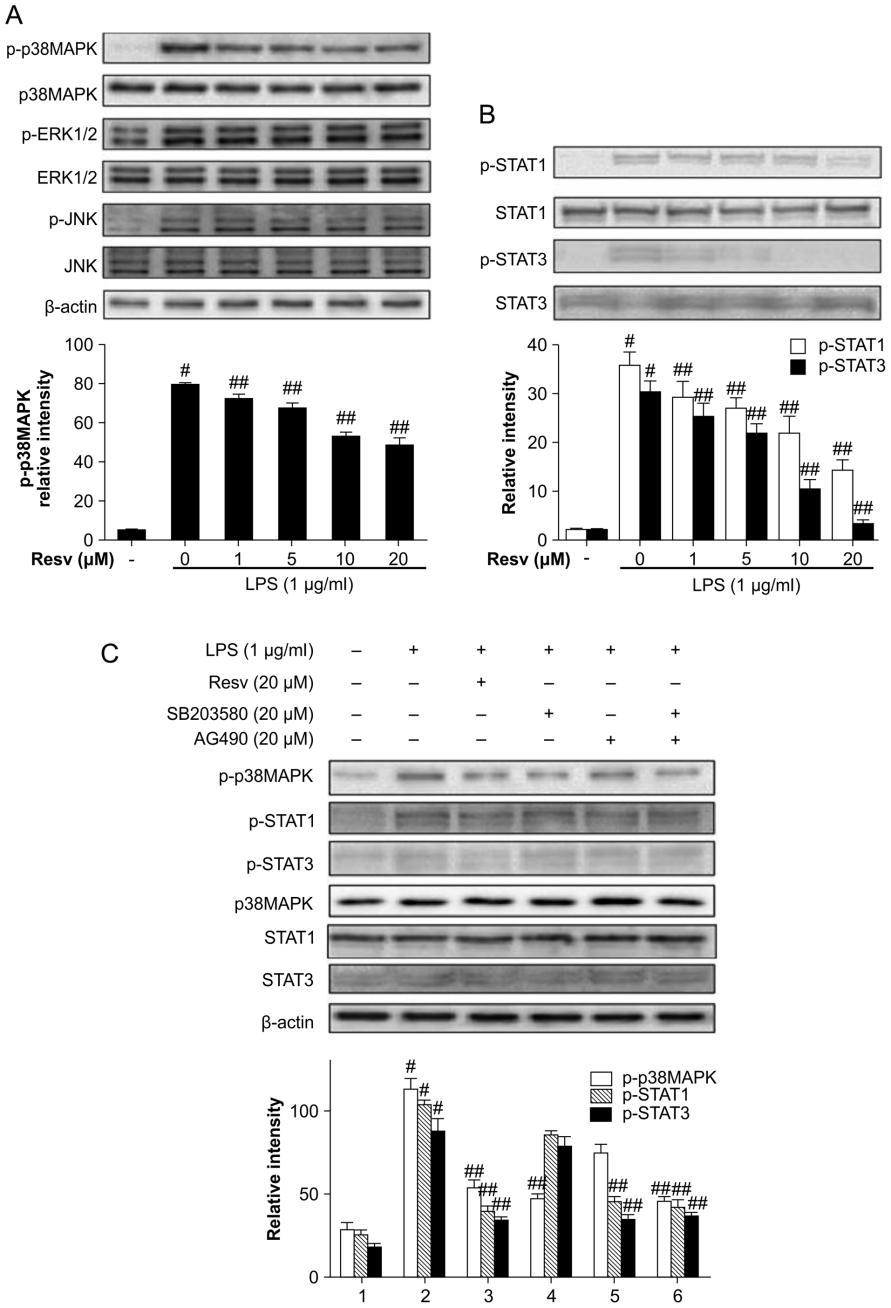
Resveratrol reduces lipopolysaccharide (LPS)-induced mitogen-activated protein kinase (MAPK) and signal transducer and activator of transcription (STAT) activity. Cells were pre-treated with resveratrol for 1 h and then stimulated with 1 *μ*g/ml LPS for (A) 30 min or (B) 2 h. (C) The cells were pre-treated with 20 *μ*M resveratrol, SB20350, or AG490 for 1 h and then stimulated with LPS for 30 min and 2 h. Western blot analysis was performed with the indicated antibodies. Bands were quantified by densitometry and the results are presented as the means ± SD of 3 independent experiments. ^#^P<0.01 vs. blank control; ^##^P<0.01 vs. LPS group. Blank control, untreated cells; LPS group, cells stimulated with only LPS.

